# Immunomodulatory effects of preparations from Anthroposophical Medicine for parenteral use

**DOI:** 10.1186/s12906-015-0757-6

**Published:** 2015-07-09

**Authors:** Carsten Gründemann, Christoph Diegel, Barbara Sauer, Manuel Garcia-Käufer, Roman Huber

**Affiliations:** Center for Complementary Medicine, Institute for Environmental Health Sciences, University Medical Center Freiburg, Breisacher Str. 115B, 79106 Freiburg, Germany

**Keywords:** Inflammation, Rheumatoid arthritis, Bryophyllum, *Kalanchoe pinnata*, *Colchicum officinale*, *Mandragora officinale*, *Rosmarinus officinale,* lymphocytes, dendritic cells

## Abstract

**Background:**

Preparations from anthroposophical medicine (AM) are clinically used to treat inflammatory disorders. We wanted to investigate effects of a selection of AM medications for parenteral use in cell-based systems *in vitro*.

**Methods:**

Colchicum officinale tuber D3, Mandragora D3, Rosmarinus officinale 5 % and Bryophyllum 5 % were selected for the experiments. Induction of apoptosis and necrosis (human lymphocytes and dendritic cells [DCs]) and proliferation of lymphocytes as well as maturation (expression of CD14, CD83 and CD86) and cytokine secretion (IL-10, IL12p70) of DCs were analyzed. Furthermore, proliferation of allogeneic human T lymphocytes was investigated *in vitro* in coculture experiments using mature DCs in comparison to controls.

**Results:**

The respective preparations did not induce apoptosis or necrosis in lymphocytes or DCs. Lymphocyte proliferation was dose-dependently reduced by Colchicum officinale tuber D3 while the viability was unchanged. *Rosmarinus officinale* 5 %, but not the other preparations, dose-dependently inhibited the maturation of immature DCs, reduced secretion of IL-10 and IL-12p70 and slightly inhibited proliferation of allogeneic CD4^+^ T-lymphocytes in coculture experiments with DCs.

**Conclusion:**

The selected preparations from AM for parenteral use are nontoxic to lymphocytes and DCs. *Rosmarinus officinale* 5 % has immunosuppressive properties on key functions of the immune system which propose further investigation.

## Background

Anthroposophical medicine (AM) is a whole medical system comprising, among others, art therapies, movement therapies (therapeutic eurythmy) and over 2,000 different medications [1, 2] based on natural substances (minerals, plants, animals) in homeopathic dilution or as undiluted extracts. Most of these drugs used in AM are officially registered in Germany and Switzerland and are available for clinical use in most European countries.

Hundreds of these drugs are manufactured in ampoules and approved for parenteral (subcutaneous and/or intravenous) use. They are clinically applied for the treatment of various diseases. In our outpatient center, which is visited by approximately 2,000 patients per year, we have seen good outcomes in patients with inflammatory diseases during treatment with AM parenteralia. We were therefore interested in knowing whether parenterally applied medications from AM which are used to treat inflammatory related disorders like rheumatoid arthritis, gout or diabetes [3] have immunomodulatory properties on healthy immunocompetent cells *in vitro*.

From the over 2,000 different medications of AM listed in [1, 2], we randomly selected four which fulfilled all of the three following criteria: I.) availability for parenteral use, II.) availability in substantial concentrations i.e. not homoeopathically diluted > D3 (1:1000) and III.) having documented use for treatment of inflammatory disorders. Based on these criteria, we selected the highest concentrations available of *Colchicum officinale* (Colchicum tuber D3), *Mandragora officinale* (Mandragora root D3), *Rosmarinus officinale* (Rosemary leaves 5 %) and *Kalanchoe pinnata* (Bryophyllum leaves 5 %). All medications have not been investigated in such experiments before.

Colchicum as Colchicine standardized extract is used for gout treatment in conventional medicine [4]. The alkaloid colchicine inhibits mitosis and prevents granulocytes from invading the gout-affected joint. *In vitro* and in patients, it has shown to induce lymphocyte and monocyte activation as well as the proliferation of mitogen-activated lymphocytes [5–7], which resulted in clinical trials with patients suffering from asthma, liver fibrosis and others [8–10]. For familial Mediterranean fever, Behçet's disease and pericarditis with effusion, its efficacy has been recognized [10]. Colchicum tuber is also used in AM for treating gout [3].

Indications for Mandragora use in AM are mainly painful, degenerative and inflammatory disorders of joints and ligaments. It has been used in folk medicine as a narcotic, an anesthetic and for the treatment of various diseases [11, 12] and contains alkaloids with anticholinergic properties.

Rosemary 5 % is listed in an AM textbook as supportive treatment for diabetes [3]. Phenolic compounds from rosemary and extracts from rosemary exerted in *in vitro* studies anti-inflammatory [13, 14] and anti-diabetic [15, 16] properties.

Bryophyllum 5 % is clinically used in AM to improve restlessness and tension-related symptoms—e.g., in premature labor [17], but also in patients with diabetes or hyperthyroidism [3] in order to reduce inflammation. The extract has shown smooth muscle relaxing properties [18, 19]. Other extracts from the Bryophyllum family than *Kalanchoe pinnata* have shown anti-diabetic activity [20] and anti-inflammatory effects [21, 22].

Because of the use of drugs from AM in inflammatory disorders and the lack of previous experiments, we investigated the immunomodulatory potential of a selection of these preparations in cell-based systems. Lymphocytes and dendritic cells (DCs) were used as model systems. As outcome criteria, we determined lymphocyte viability to see whether it is possible that the immunomodulatory effects of the extracts are mediated through cytotoxicity and lymphocyte proliferation to get a hint on immunosuppressive or immunostimulating properties.

Maturation and function of DCs were analyzed because DCs play a key role in regulating the immune function of the body. Their functions are phagocytosis, antigen recognition and processing, maturation and initiation of an adaptive immune response by presenting antigens and activating T cells. As a result, they mediate between the innate and the adoptive immune system [23]. Errant DCs are involved in the development of autoimmune diseases. Maturation of DCs is, amongst others, indicated by a shift of activation of costimulatory surface molecules (CD 83, CD 86 and CD 14), which can be measured by FACS-analyses [24].

## Methods

### Ethics statement

Patients gave their written consent for giving blood for scientific research. All experiments conducted on human material were approved by the ethics committee of the University of Freiburg (55/14) and conform to the declaration of Helsinki.

### Medication

The investigated injectable preparations are officially registered according to § 38/39 of the German Drug Law, have marketing authorization in Germany and are available in the European Union. The extracts from Colchicum and Mandragora are manufactured according to methods 21 and 19f respectively of the German Homeopathic Pharmacopoeia [25]. All specifications for parental medications were fulfilled according to the European Pharmacopoeia (EP) [26]. Good Manufacturing Practise (GMP) and quality control according to the EP is monitored from the German authorities (Federal Institute for Drugs and Medical Devices; BfArM). This includes proof of plant source identity and absence of contamination with heavy metals, pesticides, aflatoxins and microorganisms. Ampoules from the sales stock were sent to our laboratory in Freiburg, Germany, where the cell biological experiments were performed. For each experiment, a fresh ampoule was used.

Colchicum officinale tuber D3 is manufactured from a watery extract of the bulb of the flowering meadow saffron. The mother extract is three times diluted 1:10 (D3) in physiological saline according to the homeopathic pharmacopoeia. For Mandragora D3, 10 parts of dried Mandragora root is mixed with 100 parts of ethanol 43 %, mixed and boiled for 30 min. Afterwards, the extract is pressed and sterile filtered. This mother tincture is defined as D1. The D3 dilution is prepared by dilution 1:100 with physiological saline. For Rosmarinus 5 %, 10 parts of dried rosemary leaves were mixed with 100 parts of sterile water and were warmed for 5 minutes at 90 °C. The extract was pressed and filtered and was defined as D1 solution. One ampoule (1 mL) contains 0.5 mL of this rosemary extract and 0.5 mL physiological saline. One ampoule (1 mL) of Bryophyllum 5 % contains 0.1 g of a watery extract from *Kalanchoe pinnata* leaves (1:1.1) and physiological saline. All extracts are manufactured and marketed from Weleda AG (Schwäbisch Gmünd, Germany) and sterile filtered before filling 1 mL ampoules.

### Selection of human peripheral lymphocytes and purified CD4^+^ T cells

Human peripheral lymphocytes (PBMC) were isolated from the blood of healthy adult donors obtained from the Blood Transfusion Centre (University Medical Center, Freiburg, Germany). Venous blood was centrifuged on a LymphoPrep^TM^ gradient (density: 1.077 g/cm^3^, 20 min, 500 x g, 20 °C; Progen, Heidelberg, Germany). Cells were washed twice with PBS (Life Technologies, Darmstadt, Germany), and cell viability as well as cell concentration were determined using the trypan blue exclusion test. Purified untouched T cells were obtained by CD4^+^ negative selection using the magnetic cell separation method. Cell suspension of 10^8^/mL was prepared and 100 μL of the EasySep® Positive Selection Cocktail was added following an incubation time of 10 minutes at room temperature. Afterwards, 50 μL/mL of the magnetic nanoparticles were added, well mixed and incubated at room temperature. After 5 minutes, the cell suspension was fixed to a total volume of 2.5 mL by adding recommended medium. The cells were fixed in the EasySep® magnet for 5 minutes and afterwards the supernatant was discarded and cell number was determined (all products from StemCell Technologies, Grenoble, France). Cells were cultured in RPMI 1640 full media (supplemented with 10 % heat-inactivated fetal calf serum (PAA, Pasching, Austria), 2 mM L-glutamine, 100 U/mL penicillin and 100 U/mL streptomycin (all from Life Technologies, Darmstadt, Germany). The cells were cultured at 37 °C in a humidified incubator.

### Generation and maturation of immature monocyte-derived DCs

Immature DCs (iDCs) were generated by plastic adherence or CD14-positive selection method. For plastic adherence, isolated PBMC were cultured in RPMI 1640 media (Life Technologies, Darmstadt, Germany) supplemented with 2 % human albumin (Albunorm 20 %; Octapharma, Langenfeld, Germany) at a concentration of 2x10^6^ cells/cm^2^ and cultured for adherence of monocytes for 2 hrs at 37 °C in a 5 % CO_2_/95 % air atmosphere. The nonadherent cells were carefully removed by washing twice with PBS. Purified CD14^+^ monocytes were obtained by CD14^+^ positive selection. Cell suspension of 10^8^/mL was prepared, and 100 μL of the EasySep® Positive Selection Cocktail was added following an incubation time of 10 min at room temperature. Afterwards, 50 μL/mL of the magnetic nanoparticles were added, well mixed and incubated at room temperature. After 5 minutes, the cell suspension was fixed to a total volume of 2.5 mL by adding the recommended medium. The cells were fixed in the EasySep® magnet for 5 minutes, and afterwards, the supernatant was discarded and cell number was determined (all products from StemCell Technologies, Grenoble, France). For generation of iDCs, the cells were cultured in serum-free CellGro DC Media (CellGenix, Freiburg, Germany) added with 800U/mL recombinant human IL-4 (PeproTech, Hamburg, Germany) and 1000U/mL recombinant human GM-CSF (Leukine sargramostim; Bayer, Leverkusen, Germany) for 5–6 days at 37 °C in a 5 % CO_2_/95 % air atmosphere. For maturation, iDCs were harvested and were further cultivated at a density of 4x10^5^ cells/mL for 24–48 hrs in RPMI 1640 full media in the presence of media or maturation cocktail (500 ng/mL LPS (Sigma-Aldrich, Taufkirchen, Germany); 50 ng/mL TNF-alpha and 10 ng/mL IL-1beta (both from PeproTech, Hamburg, Germany) alone or supplemented with different concentrations of anthroposophical test medications or controls as indicated.

### Determination of lymphocyte and DC apoptosis and necrosis using annexin V and propidium iodide staining

Biological effects of plant-derived extracts on cells are often mediated through cytotoxicity. Therefore, the levels of apoptosis and necrosis were determined using the annexin V-FITC apoptosis/necrosis detection kit (eBioscience, Frankfurt, Germany). In detail, cells were stained for 15 minutes at room temperature in the dark with an annexin V antibody. Afterwards, propidium iodide was added to the wells, and cells were further incubated for 10 minutes. After staining, cells were analyzed by flow cytometry to determine the amount of apoptotic and necrotic cells. Positive controls for apoptosis and necrosis CPT (100 μM) and Triton-X 100 (0.5 %) were used, respectively. Whole cell population was gated for analysis.

### Cell division tracking of lymphocytes using CFSE staining

PBMC were harvested and washed twice in cold PBS before they were resuspended in PBS at a concentration of 5 × 10^6^ cells/mL. CFSE (carboxyfluorescein diacetate succinimidyl ester, 5 mM; Sigma, Taufkirchen, Germany) was added in 1/1000 dilution, and the PBMC were incubated for 10 min at 37 °C. The staining reaction was stopped by washing twice with complete medium. Afterwards, the cell division progress was analyzed using flow cytometry.

### Surface receptor analysis and cytokine determintation of cendritic cells

The effects of anthroposophical medications on DC maturation were determined by measuring surface receptor expression (anti-human CD14, CD83 and CD86 mABs; all from ebioscience, Frankfurt, Germany) using live cell gating in flow cytometric analysis. IL-10 and IL-12p70 were detected and analyzed in the supernatants of cultured cells using classical ELISA technique (ebioscience, Frankfurt, Germany). The detection levels were 2 and 4 pg/mL respectively.

### Cocultivation of DCs and allogeneic purified T cells

For cocultivation experiments, purified CD4^+^ T cells (as described in selection of human peripheral lymphocytes and purified CD4^+^ T cells) were harvested and washed twice in cold PBS and resuspended in PBS at a concentration of 5x10^6^ cells/mL. Cells were incubated for 10 min at 37 °C with carboxyfluorescein diacetate succinimidyl ester (CFSE; 5 μM: Sigma-Aldrich, Taufkirchen, Germany). The staining reaction was stopped by washing twice with complete RPMI 1640 media. The CFSE^+^ CD4^+^ T cells (5x10^5^) were cultured in 96 U-bottomed plates (Greiner, Frickenhausen, Germany) with mature DCs (mDCs) (5x10^4^) that had been matured in the presence or absence of different concentrations of Rosmarinus officinale 5 % extract. For analyzing T cell proliferation, the DC:T cell ratio of 1:10 was used, and cells were cultured for 5 days, followed by flow cytometric analysis. As control, CFSE^+^ CD4^+^ T cells were cultured with media alone.

### Data analysis

For statistical analysis, data were processed with Microsoft Excel and SPSS software (IBM, Version 22.0, Armonk, USA). Values are presented as mean ± SD for the indicated number of independent experiments. As a preliminary point in statistical analysis, normality of data was confirmed by the Shapiro-Wilk test. Statistical significance was determined by one-way ANOVA followed by Dunnett's post hoc pairwise comparisons or by paired two-sample *t*-tests. The asterisks represent significant differences from the control group (*P < 0.05, **P < 0.01, ***P < 0.001).

## Results

### Injectable preparations of AM have no effect on apoptosis and necrosis induction of lymphocytes and DCs

**Fig. 1:**
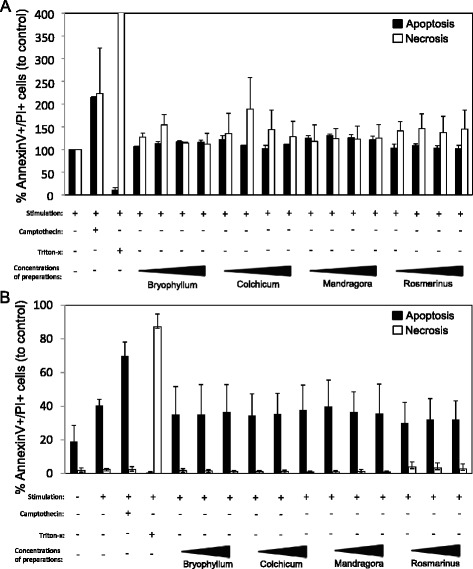
Effects of injectable preparations on apoptosis and necrosis of lymphocytes and DCs. After stimulation of lymphocytes (**a**) or DCs (**b**) with antihuman CD3/28 mAbs (each 10 ng/mL) or TNF-alpha, LPS and IL-1beta, respectively, in the presence of medium, camptothecin (100 μM), Triton X-100 (0.5 %) or different concentrations of Bryophyllum, Colchicum and Mandragora (all 1:300 (only in A); 1:100; 1:30; 1:10) or Rosmarinus (1:400 (only in A); 1:200; 1:100; 1:50). The cells were stained with annexin V and propidium iodide (PI) to assess the percentage of apoptotic (annexin V^+^/PI^−^) and (annexin V^+^/PI^+^) or necrotic (annexin V^−^/PI^+^) cells. The cells were analysed by flow cytometry. The results from two to three independent experiments are summarized, and data are presented as mean ± standard deviation (SD)

We first investigated the effect of four different anthroposophical preparations on apoptosis and necrosis induction of lymphocytes (Fig. 1a) and DCs (Fig. 1b). In the combined annexin V/propidium iodide flow cytometric analysis, the used concentrations of Bryophyllum, Colchicum, Mandragora and Rosmarinus did not induce apoptosis or necrosis of lymphocytes or DCs compared to controls.

### Injectable Colchicum and Rosmarinus preparation affect lymphocyte proliferation

**Fig. 2:**
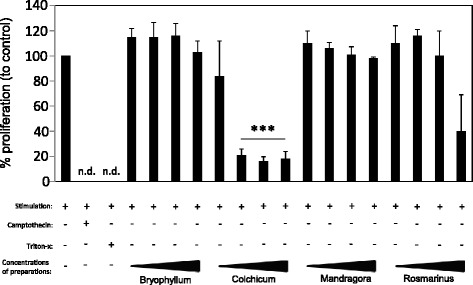
Effects of injectable preparations on proliferation of lymphocytes. CFSE^+^ lymphocytes were activated with antihuman CD3/28 mAbs (each 10 ng/mL) and were treated with medium, camptothecin (100 μM), Triton X-100 (0.5 %) or different concentrations of Bryophyllum, Colchicum and Mandragora (all 1:300; 1:100; 1:30; 1:10) or Rosmarinus (1:400; 1:200; 1:100; 1:50) for 72 hrs. After cultivation, the cell division was investigated using flow cytometric analysis. The results from three independent experiments are summarized, and data are presented as mean ± standard deviation (SD). The asterisks represent significant differences from untreated, activated lymphocytes alone. n.d. = not detectable (***P < 0.001)

We further analysed the impact of different concentrations of Bryophyllum, Colchicum, Mandragora and Rosmarinus preparations on the cell division of activated lymphocytes (Fig. 2) and observed that the Bryophyllum and Mandragora preparations have no impact on the proliferation of lymphocytes. Cells treated with different concentrations of Colchicum inhibited the proliferation of lymphocytes in the used concentration range. Rosmarinus diminished this parameter only at a high concentration.

### Injectable Rosmarinus preparation affects maturation of DCs

**Fig. 3:**
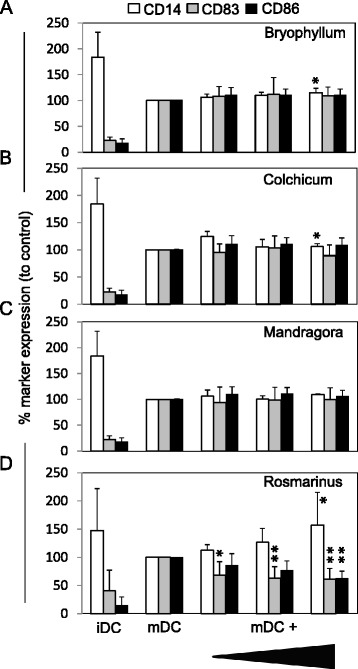
Effects of injectable preparations on DC maturation markers. mDC were cultured in the presence of different concentrations of Bryophyllum (**a**; 1:300; 1:100; 1:30), Colchicum (**b**; 1:300; 1:100; 1:30), Mandragora (**c**; 1:100; 1:30; 1:10) or Rosmarinus (**d**; 1:200; 1:100; 1:50) for 48 hrs, and marker expression of CD14 (white bars), CD83 (grey bars) and CD86 (black bars) were detected by using respective mAbs in flow cytometric analysis. As reference, iDCs were stained with mAbs in parallel. Results were shown from four to six independent experiments, and data were presented as mean ± standard deviation (SD) in relation to mDCs (=100 %). The asterisks represent significant differences from untreated mDCs alone (*P < 0.05; **P < 0.01)

In the next step, we characterized the effects of Bryophyllum, Colchicum, Mandragora and Rosmarinus (Fig. 3) on expression of typical markers (CD14, CD83 and CD86) associated with the maturation status of DCs. As shown in Fig. 3a-d, iDCs express high amounts of CD14 and low amounts of the surface markers CD83 and CD86. After activation with TNF-alpha, LPS and IL-1beta, CD14 was down-regulated, and the expression of CD83 and CD86 increased on the surface of the cells. The used concentrations of Bryophyllum, Colchicum and Mandragora (Fig. 3a-c) had no influence on marker expression, but incubation with different concentrations of Rosmarinus (Fig. 3d) inhibited the maturation of DCs. CD14 expression increased (0.25 mg/mL: 113 % ± 10; 0.5 mg/mL: 127 % ± 25; 1.0 mg/mL: 157 % ± 58), while CD83 expression (0.25 mg/mL:: 69 % ± 19; 0.5 mg/mL: 63 % ± 21; 1.0 mg/mL: 61 % ± 24) as well as CD86 expression (0.25 mg/mL: 86 % ± 12; 0.5 mg/mL: 77 % ± 17; 1.0 mg/mL: 63 % ± 20) decreased compared to mDCs incubated with media alone (=100 %).

### Injectable Rosmarinus preparation modulates cytokine secretion of DCs

**Fig. 4:**
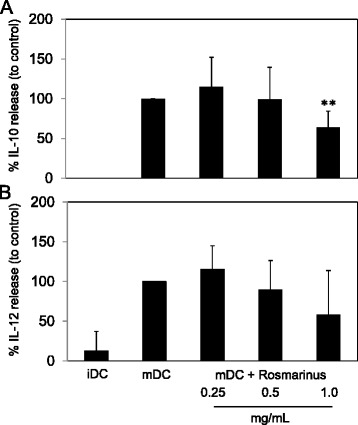
Effects of Rosmarinus injectable preparation on cytokine release of DCs. Secretion of IL-10 (**a**) and IL-12p70 (**b**) of iDCs or mDCs alone or in the presence of different concentrations of Rosmarinus (0.25-1 mg/mL) were analyzed in the supernatants of the cells using ELISA-based flow cytometry. Results are summarized from four to six independent experiments, and data are presented as mean ± SD. The detection levels were 2 pg/mL (IL-10) and 4 pg/mL (IL-12) respectively. The asterisks represent significant differences from untreated mDCs alone (**P < 0.01; paired t-test)

We cultured DCs in the presence of different concentrations of Rosmarinus (1:2000–1:500 corresponding to 0.25- 1 mg/mL) to see whether there is an influence on IL-10 and IL12p70 secretion of DCs (Fig. 4). iDCs per se did not secrete IL-10 (Fig. 4a; no detection) and IL-12 (Fig. 4b; 12 % ± 25) in comparison to mDCs (=100 %). The presence of the Rosmarinus preparation reduced the release of IL-10 (Fig. 4a) (0.25 mg/mL: 115 % ± 37; 0.5 mg/mL: 99 % ± 40; 1.0 mg/mL: 64 % ± 21) and IL12p70 (Fig. 4b) (0.25 mg/mL: 115 % ± 30; 0.5 mg/mL: 89 % ± 37; 1.0 mg/mL: 58 % ± 56) which shows that not only maturation but also the activation status of DCs is modulated by the Rosmarinus extract.

### Injectable Rosmarinus preparation inhibits the proliferation of allogeneic CD4^+^ T cells

**Fig. 5:**
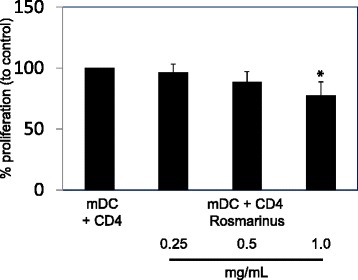
Effects of Rosmarinus injectable preparation on proliferation of allogeneic CD4^+^ T cells. mDCs were cocultured with allogeneic CFSE-labelled purified human CD4^+^ T cells (CD4) in the presence of different concentrations of Rosmarinus (0.25-1 mg/mL) using a DC:T cell ratio of 10:1 for 5 to 6 days. Cell division analysis was assessed by flow cytometry and data are presented as mean ± SD of three independent experiments. The asterisks represent significant differences from untreated CD4^+^ T cells alone (*P < 0.05)

DCs are an important part of the immune system. Therefore, we investigated whether DCs that were generated in the presence of the Rosmarinus preparation influenced the proliferation of allogeneic purified human lymphocytes (Fig. 5). CD4^+^ T cells were purified, labelled with CFSE and were activated with allogeneic DCs in the presence of media or different concentrations (0.25- 1 mg/mL) of the Rosmarinus preparation. The CFSE dye is inherited by daughter cells after cell division and each dividing cell loses fluorescent intensity measured by flow cytometry. The data revealed that allogeneic CD4^+^ T cells (CD4) proliferate in the presence of mDCs. Furthermore, DCs which were preincubated with different concentrations of the Rosmarinus extract inhibited the proliferation of allogeneic CD4^+^ T cells only at high concentrations.

## Discussion

Preparations from AM have been developed from an holistic view on nature and spiritual science [3] and not from bench to bedside. Thus, it can be explained that these preparations are clinically used for the treatment of inflammatory disorders but have not yet been tested regarding effects on cells of the human immune system. Marketing authorisation in Germany and Switzerland results from history.

Lymphocytes and DCs were used as model systems because they are important in regulating immunity and preventing autoimmune diseases. Because we wanted to evaluate the selected preparations as such, we abstained from separately testing the effects of known immunomodulating compounds in relation to the extracts. Especially for the Colchicum extract, a relation to a known immunomodulating compound, namely Colchicine, could be expected. Colchicine inhibits mitosis and proliferation of immunocompetent cells [5–7] and was present in the used Colchicum tuber extract in related doses because Colchicum tuber plant material for pharmaceutical use is defined to have 0.05–0.4 % Colchicine according to German regulations [12]. The inhibition of lymphocyte proliferation by the Colchicum tuber extract used in AM is, therefore, plausible.

Rosmarinus and Bryophyllum are the only parenteral preparations of AM which are available in the concentration of 5 %. While Bryophyllum 5 % does not affect the immune response, Rosmarinus 5 % clearly inhibited maturation and functionality of DCs. Because this effect is not mediated by apoptosis or necrosis, its molecular mechanism and the clinical implications for the treatment of diseases with an overactive immune system deserve further investigation. The concentrations used in the *in vitro* experiment are comparable to the concentrations which are reached locally after subcutaneous injection of one ampoule of the respective preparations. Systemic concentrations in the human body cannot be estimated because the distribution volume and the distribution velocity are not known for the preparations.

## Conclusions

Differential effects of the different AM medications on immune function have been found. Colchicum has immunosuppressive properties on cell proliferation of lymphocytes, while Mandragora D3, and Bryophyllum 5 % do not. Rosmarinus officinale 5% additionally inhibited functions of DCs. Clinical relevance and molecular mechanisms of these findings should be further evaluated.
